# Why Do Adolescents Use Fluoride Toothpaste? A Qualitative Interview Investigation

**DOI:** 10.3290/j.ohpd.a44681

**Published:** 2020-07-04

**Authors:** Kristina Furundzic, Joy Malmberg, Boel Sandström, Dan Ericson

**Affiliations:** a Dentist, Skåne County Public Dental Service, Malmö, Sweden. Contributed to study design, performed the interviews, acquisition of data and primary analysis of data, writing and revising the manuscript critically for important intellectual content, agreed on the final version of the article.; b Dentist, Department of Cariology, Faculty of Odontology, Malmö University, Malmö and Skåne County Public Dental Service, Trelleborg, Sweden. Contributed to study design, performed the interviews, acquisition of data and primary analysis of data, wrote the manuscript, revised it critically for important intellectual content, agreed on the final version of the article.; c ^Senior^ Lecturer, Department of Health, Blekinge Institute of Technology, Karlskrona, Sweden. Contributed to study design, interpreted data, wrote the manuscript, revised it critically for important intellectual content, agreed on the final version of the article.; d Professor, Department of Cariology, Faculty of Odontology, Malmö University, Malmö, Sweden. Contributed to the design, wrote the manuscript and revised it critically for important intellectual content, agreed on the final version of the article.

**Keywords:** adolescents, fluoride toothpaste, oral health behavior, motivation, qualitative research

## Abstract

**Purpose:**

Fluoride toothpastes are effective in caries prevention. In legislation, regular fluoride toothpaste is a cosmetic product; adolescents use it for aesthetic purposes. In dentistry, fluoride toothpaste is considered a caries preventive drug recommended to patients for that reason. Knowledge is lacking concerning what motivates adolescents to use fluoride toothpaste. Dental professionals need to understand how to motivate a risk-group for caries development to use fluoride toothpaste frequently in order to effectively motivate patients to prevent tooth decay. The purpose of this study was to investigate what motivates adolescents to use fluoride toothpaste.

**Materials and Methods:**

The study was conducted at a high school in southern Sweden. The final sample consisted of 16 adolescents age 16 to 19. This study employed a qualitative design using semi-structured interviews. The data were analysed using manifest content analysis with an occasional inductive approach.

**Results:**

Reasons for why adolescents use fluoride toothpaste were found in four different categories: oral health, economy, upbringing and habit, social influences.

**Conclusion:**

There are reasons to believe that dental professionals might have missed important arguments for why adolescents use fluoride toothpaste. The participants mentioned oral health and aesthetics as important reasons for using fluoride toothpaste, as well as other more surprising factors such as financial reasons and social environment. There are thus more arguments for using fluoride toothpaste that adolescents value than the ones we believe dental professionals use.

Fluoride toothpaste is globally advertised largely from a cosmetic perspective. This conforms to the legal definition of regular fluoride toothpaste as a cosmetic product^4^ and adolescents indeed use toothpastes for cosmetic purposes.^3^ Dental professionals, on the other hand, promote fluoride toothpaste for its therapeutic effect in caries prevention.^23^ An increasing number of children and adolescents request tooth bleaching.^3^ This coincides with a general commercial focus on aesthetics. However, it is uncertain what motivates adolescents to use fluoride toothpaste: cosmetics or caries prevention. If dental professionals knew what motivates adolescents in this respect, they might be able to better understand what arguments to use to motivate adolescents to use fluoride toothpaste.^22,23^

In the UK (2015) parents were the dominant influence on attitudes toward oral health behaviour, which implies that oral health strategy ought to engage parents.^9^ The dentist was seen as a supporter of the oral health behaviour advocated by the parents. With time, parental influence became less important and the external environment – including the availability of sugar-rich food and peer influence – became more relevant. The adolescents believed that oral and general health were equally important and closely interlinked, but also acknowledged the social impact of an aesthetic smile.

The knowledge about fluoride’s preventive effects was generally low also in a Swedish population, and the driving force behind oral hygiene procedures was a pleasant feeling and social norms, as well as (with increasing age) factors related to health and disease prevention.^11^ Toothbrushing was considered equal in important to fluoride for caries prevention.^12^

Oral health professionals in Sweden seem to lack knowledge, time, and attitude, which complicates oral health recommendations. Professionals generally thought it was easier to highlight plaque on teeth to their patients than to ask about their use of fluoride toothpaste. The reason was that the professionals assumed that patients already had the necessary knowledge of fluoride toothpaste and its use.^6,13^ Nevertheless, individually-based intervention by a dental hygienist has a significant impact on the use of fluoride tooth paste.^14^

Therefore, it is highly relevant to reveal what motivates adolescents to use fluoride toothpaste in order to develop appropriate arguments.

## Materials and Methods

### Ethics Approval and Consent to Participate

The study was approved by the local ethics committee of Malmö University, Faculty of Odontology local ethics (Study 3.5.3-2016/506). Permission to conduct the study at the selected high school was approved by the principal. Participants were informed of their right to withdraw their participation at any time and without any consequences. Informed consent was collected, and parents of minors gave their approval for their children’s participation. All materials were handled confidentially. The interviews were coded in numbers and it was not possible to identify the participants in the study. Digital audio recordings were destroyed at the end of the study.

### Study Population Background

The Swedish Dental Act states that all citizens of Sweden have the right to good quality dental care and the ambition is that all citizens should receive dental care on equal terms.^21^ This means that information about a patient’s dental status, treatments, or information about prophylaxis should be adapted to the patient’s age, maturity, experience, language, and other individual conditions according to the Patient Act.^20^

### Study Design and Sampling Procedure

This study used data collected from an open question in a questionnaire from a previous study. The answers to the question “Describe a situation where you can experience the advantage of using or having used toothpaste” were analysed by using manifest content analysis.^2^ The analysis resulted in eight themes, serving as a basis for the interview guide for this study.

Since the aim of this study was to investigate why adolescents use fluoride toothpaste, with the aim of understanding how subjects in a specific environment interpret their social reality, a qualitative design with semi-structured interviews was conducted.^1,2^

The study took place at a public high school in a city in southern Sweden with 880 students from different districts. The school had three programmes: a Natural Science programme, a Social Science programme and an Introduction programme for newly arrived students. The study information was sent to 18 teachers in a total of 12 classes, six within the Natural Science Programme and six within the Social Science Programme. Their teachers gave students written and oral information. The authors contacted interested students by e-mail or phone. All students chose to be interviewed at their own school except one who chose to be interviewed at the Faculty of Odontology.

The inclusion criteria were adolescents between 16 and 19 years old. A total of 18 students were interested in participating. Two of the students could not be interviewed due to planning difficulties. The final sample consisted of 16 adolescents. The gender ratio was not controlled since gender was not of interest in the analysis process. The sample was considered heterogeneous since it consisted of students of different genders and backgrounds.^10,19^ Prior to the interviews, students filled in a consent form. Students below the age of 18 had to inform their parents and receive written consent from them. Participants received a cinema ticket as thanks for participating.

### Data Collection

Qualitative semi-structured interviews were used to collect data.^2^ The interviews were based on an interview guide that was structured around the eight themes and contained a group of questions. Each of the interviews started with the question whether the adolescent used toothpaste, and if they answered “yes”, they were asked if the toothpaste contained fluoride. All answered “yes”, and they were then asked to elaborate on why and how they used it. The guide was tested and confirmed in four pilot interviews with adolescents.

The interviews were conducted in separate rooms and a quiet environment. One interviewer performed the interview and operated the digital audio recorder while the other took notes and asked additional questions when needed. The two authors (K.F. and J.M.) conducted eight interviews each. The sessions were recorded on a tape recorder and varied from five to 15 min per interview. Records were transcribed directly after the interviews.

### Data Analysis

The data were analysed using manifest content analysis with an occasional inductive approach as described by Graneheim and Lundman.^7^ The analysis was performed the same day or in some cases the day after the interviews. The transcripts were read through to gain understanding of the whole sample, then re-read several times and coded. Then, codes were analysed based on similarities and differences and grouped into subcategories which were contrasted with the text to ensure credibility. Finally, the subcategories were reduced and interpreted to represent four main categories. The same two authors coded and categorised all data. Each sentence was analysed by both authors and constantly compared. If they did not agree on a code or category, alternatives were discussed until agreement was reached.

## Results

Four different categories were identified: oral health, economy, upbringing and habit, and social influences.

### Oral Health

When asked about why they were using fluoride toothpaste, most of the participants mentioned oral health to some extent. Toothpaste was described by several participants as something that makes the teeth stronger and kills bacteria and thus prevent caries. Sugar would have less effect on the teeth if one used toothpaste. One participant expressed theories about pH levels and believed that toothpaste could be used to protect against acid as toothpaste is basic and therefore increases the pH.

Different theories about what role fluoride in toothpaste played in oral health were also expressed. One participant stated that fluoride was probably the substance in the toothpaste that prevented gingivitis and another participant thought that fluoride kills bacteria inside the teeth. Other participants mentioned plaque removal and eliminating bacteria to avoid tooth decay, caries, toothache, and tooth lossi. One of the participants talked about the consequences of tooth loss: “(you should use toothpaste) to be able to chew your food in the future, for your, what is it called? ‘life-expectancy’, will be much better if you can actually get food inside of you, physically, or it will be a bit hard if you have to get it intravenously” (Interview 3).

All in all, the participants were unsure about how fluoride in toothpaste worked and some of them were willing to switch to something with the same taste, such as mouth rinse.

### Economy

Some of the statements concerned the financial aspects of using toothpaste when brushing teeth. Participants mentioned that it would be favourable to use toothpaste to prevent dental disease and tooth loss. Toothache and missing teeth were not considered to be an issue since there would always be the possibility of replacing bad teeth with artificial ones. However, the procedure was regarded as expensive and therefore supposed to have financial consequences. The participants’ experiences of their parents’ problems with getting their teeth fixed was the reason why they tried to avoid similar situations. One of the participants said that she wanted to avoid ruining her teeth since she had been receiving free dental care during her child- and adulthood. “My dad did not take care of his teeth when he was young because he did not have the opportunity and it has destroyed his teeth. It costs a lot of money and I want to avoid it, since I have received so much... The privilege of growing up in Sweden, free dental care...” (Interview 2).

### Upbringing and Habit

The analysis revealed that using toothpaste was a habit and a daily routine, and something that many of the participants just kept doing without reflecting on it. For toothbrushing to become a habit, an individual must have been brushing for a long time. Most of the participants said that it was their parents who introduced them to toothpaste and made them keep using it as they grew older, so it became a habit. Brushing their teeth with toothpaste was a natural thing to do, since they had done that for as long as they could remember. “I was introduced to it when I was very little. I think I might have been 5-6 years old when I first started using it myself. Eh, and then, like, it became a habit, I started brushing my teeth twice a day” (Interview 11).

Some also mentioned that what the dentist says about fluoride toothpaste was important, especially when the relationship with the dentist was good. One of the participants started to brush his teeth and use fluoride toothpaste just recently, after his dentist informed him about how it works. Before this, he just thought about it as using soap, but for your mouth, and as his peers and parents did not use fluoride toothpaste, there was no one around to encourage him to use it. He declared the importance of health literacy and getting that through your dentist, teacher, or friends.

### Social Influences

Social influences were significant. For some participants there was no question about the fact that the use of toothpaste when brushing your teeth was something deep-seated in their culture. People in their social environment were also seen as having a great impact and one of the participants said, “You do what others do”, “It is just something deep-rooted in our culture. No one questions it” (Interview 15).

The participants revealed that they often use toothpaste before they meet other people socially, for example, at school or parties, to make sure that they looked and felt clean and had a fresh taste and breath and thus felt comfortable speaking to others. Another reason for using toothpaste was to avoid getting yellow or ugly teeth. Some of the participants mentioned that white teeth are important in society today and using toothpaste can make your teeth less yellow. The reason for using toothpaste was that they wanted to look attractive. They also wanted to demonstrate that they took care of themselves. Unclean and ugly teeth were a sign of poor personal hygiene. “If you have worse teeth than others you show that you can’t take care of your body” (Interview 8).

The analysis showed that other people’s teeth were something some participants noticed and reflected upon. If someone for example had missing or yellow teeth and revealed it while smiling or talking, the participants focused on it and reacted to it even though they admitted that their reaction was superficial. Some participants also mentioned celebrities and people in media and commercials as a factor for why they use toothpaste.

## Discussion

The main finding in this study indicates that there are several different motives for adolescents’ use of fluoride toothpaste. The participants had both short- and long-term motivations. This demonstrates that the response to the question” why do adolescents use fluoride toothpaste?” is more complex than simply “to maintain healthy teeth”.

There are reasons to believe that dental professionals have missed important grounds for why adolescents use fluoride toothpaste. One reason to assume this is based on the National Swedish Dental Care bulletin “Advice about teeth”.^5^ The interviews also revealed that dental professionals only use long-term health-motivated arguments and not short-term arguments such as cosmetic or social reasons. None of the adolescents in this study stated that the dental professionals motivated them to use fluoride toothpaste for reasons other than dental health.

The participants in this study not only gave oral health and aesthetics as important reasons for why they use fluoride toothpaste, they also mentioned other more unexpected factors such as financial and social reasons. The participants assumed that taking care of their teeth as adolescents is easy since they have access to free dental care, similar to what was found in the UK.^9^

**Fig 1:**
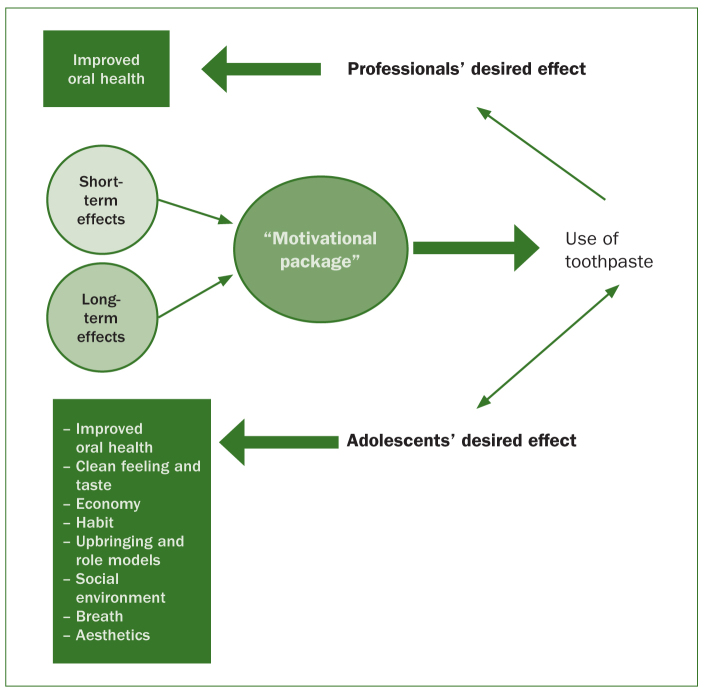
How using the motivational package will achieve both improved oral health and the adolescents’ desired effect.

All the adolescents in this study brought up long-term arguments such as good oral health, not losing teeth at a young age, and economy. But the adolescents spent most of their interview time talking about other factors that could be considered short-term reasons, such as good taste/feeling, and feeling fresh and confident in social situations. There are thus more arguments for using fluoride toothpaste that adolescents value than the ones that we believe dental professionals use. A simplified model adapted for the purpose of adolescents’ toothpaste usage was proposed (see Fig 1).

**Fig 2:**
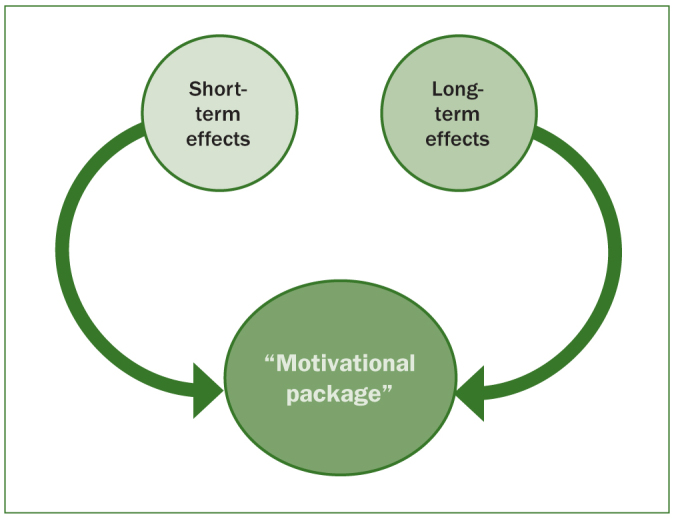
How both short- and long-term effects should make up the dental practitioners’ fluoride-toothpaste motivational package.

Since all the participants’ claims might lead to an increased use of fluoride toothpaste, we suggest the use of models demonstrated in Figs 1 and 2. Individualising the information is one more step towards good oral health and care on the same terms, which is the goal for dental care in Sweden according to the Dental Act.^21^ Jensen et al^14^ showed that individualised information could improve individuals’ oral health routines.

One might argue that dental professionals should focus on dental health and not use other arguments that might have an impact of the quality of life. But using such arguments is in line with WHO guidelines for a lower sugar intake, resulting in not only caries prevention but simultaneously preventing general health problems and a negative impact on quality of life.^18^ In this study, adolescents claimed that they used fluoride toothpaste to achieve a good taste in their mouths and before meeting other people. Therefore, the dental professionals might consider the use of such arguments, such as taste and social environment, to motivate adolescents to use fluoride toothpaste.

Healthy-looking teeth to fit in socially and to boost a positive self-image have been reported as important. Factors related to health and disease seem to become more important with age, while younger people tend to focus more on social norms.^9,11^ The adolescents in this study talked about all such factors, but the interviews tended to focus on social factors.

Qualitative investigations tend to focus on smaller samples, which makes it difficult to generalise and apply the results to other groups.^1^ Since the students in this school had above-average grades for admission to their programmes, they might differ from most adolescents.^19^ On the other hand, there were students from all over the region in this school, and knowledge about fluoride toothpaste had been gathered through information from their dentists and directly or indirectly through their parents.^15^ The results could therefore possibly be transferable to all Swedish teenagers, since they receive similar dental care. Data saturation was reached after 12 interviews.^8^ To confirm saturation, four additional interviews were conducted without any new content emerging. The sample size allowed for detailed analysis and reliable interpretation.^16^

One risk of bias was that the interviews were based on findings from a previous survey.^17^ However, reflexivity was practiced in order to prevent previous studies from affecting the result, and the immediate transcription enhanced the dependability of the study.^1^ Both authors (K.F. and J.M.) read through the transcribed text to ensure that nothing was left out and to confirm whether the interview techniques were equal.

Analysis was conducted on the day of or the day after transcription, and the authors referred to the original text during the process and discussed throughout the analysis until agreement.^1,7^ Both interviewers (K.F. and J.M.) agreed on coding the condensed meaning units into categories, thus ensuring that no relevant data was excluded.^1^ The results contain the interviewees’ voices only.^1^

A decision was made early in the process to study the group “adolescents” as a whole, since the purpose of this study was to identify reasons that adolescents have to use fluoride toothpaste. Analysis of gender and social class was not relevant to the purpose of this article.

All participants in this study used fluoride toothpaste, but for different reasons; most of them started because of their parents and then kept on using it. Habitual practices from a young age encouraged by parents seems to be an important factor.^9^ Oral health was important to most of the participants, and most of them considered that fluoride toothpaste helps achieve good oral health, even if they were not sure how it works. Knowing the details about how fluoride toothpaste works was considered less important than feeling that it is good for you.

One could argue that the sample was limited by the fact that all the participants used fluoride toothpaste. But since the previous survey revealed that all the respondents used fluoride toothpaste, the results were considered representative for the group.^6^ However, asking the respondents about how often they used fluoride toothpaste or how much fluoride toothpaste they used might have added some value.

## Conclusion

Dental professionals might have missed important arguments for why adolescents use fluoride toothpaste.^5^ The participants in this study considered not only oral health as a reason to use fluoride toothpaste, but they also focused on aesthetics and other factors, such as finances and social environment. They reported that these arguments were not mentioned by dental professionals.

All adolescents in this study mentioned some long-term reasons, for example, good oral health, not losing teeth at a young age, and economy. However, they spent most of the interview time talking about other factors that could be regarded as relevant in the short term, e.g. good taste/feeling, feeling fresh, feeling confident in social situations. There are certainly more arguments for using fluoride toothpaste that adolescents value than the ones that we believe dental professionals use.

Since all of the arguments might lead to an increased use of fluoride toothpaste, it might be beneficial to incorporate more of these arguments in dental professionals’ “fluoride toothpaste motivation package”. Individualising the information makes it more likely that the recipient understands and embraces the idea. Future research should be aimed at further investigation into what motivates adolescents and adults in different age groups to use and/or not to use fluoride toothpaste in other parts of Sweden, and perhaps internationally, to explore whether there are any differences or similarities to the results in this study. Future research may also be aimed at designing a programme to motivate patients to use fluoride toothpaste, perhaps in relation to gender and social class, and thus improve their oral health.

Since all of the arguments might lead to an increased use of fluoride toothpaste and hence improved public health, it may be recommendable to incorporate more of these arguments in dental professionals’ motivational repertoire.

## References

[ref1] Bengtsson M (2016). How to plan and perform a qualitative study using content analysis. NursingPlus Open.

[ref2] Bryman A (2015). Samhällsvetenskapliga metoder (Methods in social studies [in Swedish]).

[ref3] Donly KJ, Donly AS, Baharloo L, Rojas-Candelas E, Garcia-Godoy F, Zhou X (2002). Tooth whitening in children. Compend Cont Educ Dent.

[ref4] (2009). Europaparlamentet. Europaparlamentets och rådets förordning [EG] nr 1223/2009 den 30 november 2009 om kosmetiska produkter [omarbetning]. (The European parliaments and councils regulation [EG] nr 1223/2009 the 30th of November 2009 about cosmetic product [revision]). http://eur-lex.europa.eu/legal-content/SV/TXT/PDF/?uri=CELEX:02009R1223-20160810.

[ref5] (2016). Folktandvården Skåne. National Dental Care Skåne. Advice about teeth. https://folktandvardenskane.se/globalassets/media/pdf/info-in-foreign-languages/ftv_rad_om_tander_invandrare_engelska_tryck.pdf.

[ref6] Furundzic K, Malmberg J (2017 Apr). Vad. vet gymnasieelever om dentala erosioner? – En enkätstudie. (What do high school students know about dental erosion? – A survey study) MUEP. https://muep.mau.se/handle/2043/22491.

[ref7] Graneheim UH, Lundman B (2004). Qualitative content analysis in nursing research concepts, procedures and measures to achieve trustworthiness. Nurs Educ Today.

[ref8] Guest G, Bunce A, Johnson L (2006). How many interviews are enough? An experiment with data saturation and variability. Field Methods.

[ref9] Hall-Scullin E, Goldthorpe J, Milsom K, Tickle M (2015). A qualitative study of the views of adolescents on their caries risk and prevention behaviours. BMC Oral Health.

[ref10] Holm Ivarsson B, Sjögren K (2010). MI – motiverande samtal. Praktisk handbok för tandvården (MI – motivational interviews. Practical handbook for dental practitioners).

[ref11] Jensen O, Gabre P, Sköld UM, Birkhed D (2011). Fluoride toothpaste – Knowledge, attitudes and behaviour. Swed Dent J.

[ref12] Jensen O, Gabre P, Sköld UM, Birkhed D (2012). Is the use of fluoride toothpaste optimal? Knowledge, attitudes and behaviour concerning fluoride toothpaste and toothbrushing in different age groups in Sweden. Community Dent Oral Epidemiol.

[ref13] Jensen O, Gabre P, Sköld UM, Birkhed D, Povlsen L (2014). ‘I take for granted that patients know’ - oral health professionals’ strategies, considerations and methods when teaching patients how to use fluoride toothpaste. Int J Dent Hygiene.

[ref14] Jensen O, Moberg Sköld U, Birkhed D, Gabre P (2015). Self-reported changes in using fluoride toothpaste among older adults in Sweden: an intervention study. Acta Odontol Scand.

[ref15] Karlsson F, Wennergren R Social sortering i skolan – en studie av elevsammansättningen i Malmös gymnasieskolor (Social sorting in school – a study of pupil composition in Malmö’s high schools). https://dspace.mah.se/bitstream/handle/2043/8977/Social%20sortering%20i%20skolan.pdf?sequence=1.

[ref16] Kvale S, Brinkman S (2009). Den kvalitativa forskningsintervjun [Andra upplagan] (The qualitative research interview).

[ref17] Pannucci CJ, Wilkins EG (2010). Identifying and avoiding bias in research. Plast Reconstr Surg.

[ref18] Petersen PE (2003). The World Oral Health Report 2003: Continuous improvement of oral health in the 21st century - the approach of the WHO Global Oral Health Programme. Community Dent Oral Epidemiol.

[ref19] Skånegy. Meritvärden vid slutgiltig antagning till nationella program i Malmö 2014. 2014; (Merit ratings at final admission to national programs in Malmö). http://www.skanegy.se/sites/all/files/uploads/meritvarden_vid_slutlig_antagning_i_malmo_2014_0.pdf.

[ref20] Socialdepartementet. Patientlag [2014:821]. Svensk författningssamling 2014;821 (Swedish statutes).

[ref21] Socialdepartementet. Tandvårdslagen [1985:125] (The Dental Act [1985:125]). Svensk författningssamling 1985;985[125].

[ref22] Statens beredning för medicinsk utvärdering [SBU]. Att förebygga karies - en systematisk litteraturöversikt. (To prevent dental caries – a systematic review) SBU 2002;161.

[ref23] Statens beredning för medicinsk utvärdering [SBU]. Karies - diagnostik, riskbedömning och icke invasiv behandling. En systematisk litteraturöversikt. (Dental caries – diagnostics, risk-assessment and non-invasive treatment. A systematic review) SBU 2007;188.

